# H/ACA box small nucleolar RNA 7B acts as an oncogene and a potential prognostic biomarker in breast cancer

**DOI:** 10.1186/s12935-019-0830-1

**Published:** 2019-05-09

**Authors:** Yihan Sun, Endong Chen, Yuefeng Li, Danrong Ye, Yefeng Cai, Qingxuan Wang, Quan Li, Xiaohua Zhang

**Affiliations:** Department of Thyroid and Breast Surgery, The First Affiliated Hospital of Wenzhou Medical University, Wenzhou, 32500 Zhejiang China

**Keywords:** Small nucleolar RNA, Breast cancer, SNORA7B, Oncogenic, Apoptosis

## Abstract

**Background:**

Breast cancer (BC) is the most frequent malignancy occurring in women worldwide. Emerging evidence indicates that small nucleolar RNAs (snoRNAs) play a role in tumor development. In the current study, we evaluated expression profiles and functions of snoRNAs associated with BC.

**Methods:**

We analyzed the expression levels of snoRNAs between breast cancer and normal tissues in TCGA database and found that SNORA7B is upregulated in BC. We confirmed this result in clinical cancer tissues and BC cell lines via qRT-PCR. Then, we investigated clinical significance in public datasets and biological function of SNORA7B using a series of in vitro gain- and loss-of-function experiments.

**Results:**

SNORA7B expression was significantly upregulated in samples from patients with BC in both public database and our clinical tissues compared to its expression in normal tissues. Meanwhile, patients with high SNORA7B expression have worse prognosis. Inhibition of SNORA7B expression impaired cell growth, proliferation, migration, and invasion via inducing apoptosis.

**Conclusions:**

SNORA7B functions as an important oncogenic snoRNA in BC and may serve as a potential prognosis biomarker for BC.

**Electronic supplementary material:**

The online version of this article (10.1186/s12935-019-0830-1) contains supplementary material, which is available to authorized users.

## Background

Breast cancer (BC) is the most common malignancy occurring in women and it accounts for 30% of all diagnosed cancer in women around the world. BC remains the second leading cause of cancer-related mortality in women, especially between 20 and 59 years of age [1]. BC is a complex heterogeneous disease and has been classified into several unique molecular subtypes based on different molecular and histological characteristics [2, 3]. Although BC-related mortality has declined over the past two decades owing to great advances in cancer prevention, early diagnosis and treatment, there are still issues that need to be addressed, including micro-invasion, micro-metastasis and varied responses in patients undergoing similar surgery and adjuvant therapy [4, 5]. Routine prognostic markers such as estrogen, progesterone, and human epidermal growth factor receptors have proven to be insufficient for estimating risk of recurrence and death. Therefore, there is an urgent need to explore the complex molecular mechanisms and identify better prognostic markers to improve the life quality for patients with BC.

In recent years, small nucleolar RNAs (snoRNAs), one of the less studied classes of small non-coding RNAs of approximately 60–300 nucleotides in length, have attracted a great deal of attention as regulatory RNAs [6]. SnoRNAs originate within the intronic regions of protein-coding or non-protein coding genes and often function as housekeeping genes to guide the enzymatic modifications of other RNAs, mainly rRNA. There are two main groups of snoRNAs based on differences in structure—the box H/ACA snoRNAs (SNORAs), which are associated with pseudouridylation of rRNA, and box C/D snoRNAs (SNORDs), which are involved in 2′-*O*-methylation of rRNA [7–9]. Apart from the traditional function of modifying other RNAs, compelling evidence suggests that dysregulation of snoRNAs can also influence the development and progression of various human diseases such as Prader Willi syndrome, some metabolic stress disorders and several types of cancers [10–14]. The first report that highlighted the pathological importance of snoRNAs showed that H5sn2 (a box H/ACA snoRNA) was distinctly down-regulated in meningiomas [15]. Further, SNORD50 was reported to have a tumor suppressive role in breast and prostate cancer [16, 17], while SNORA42 was reported to act as an oncogene in lung and colorectal cancer [18, 19]. Su et al. have demonstrated the importance of snoRNAs in breast cancer [20]. With the advance of high-throughput RNA-sequencing and microarray-based analysis, snoRNAs are beginning to be considered as plausible disease biomarkers.

In the present study, we used a series of bioinformatics analysis and identified SNORA7B as a potential oncogene in BC. In order to further investigate its potential role, we performed a series of gain- and loss-of-function experiments in vitro and evaluated the relationship between the expression level of SNORA7B and clinicopathological parameters. This is the first study to explore the role of SNORA7B in BC pathogenesis.

## Materials and methods

### Patients and samples

This study analyzed 1077 patients with breast cancer and 104 patients with non-cancerous tissues from The Cancer Genome Atlas (TCGA) database. In addition, 30 pairs of matched breast cancer and adjacent normal tissues, which came from BC patients enrolled at the First affiliated hospital of Wenzhou Medical University, were used to further verify the SNORA7B expression level in BC. The use of all tissues samples in this study was approved by the Ethics Committee of the First Affiliated Hospital of Wenzhou Medical University, and informed consent was obtained from each patient.

### Cell culture

Breast cancer cell lines (MDA-MB-231, MDA-MB-468, MDA-MB-453, MDA-MB-436, MCF7, BT549, BT474, and SK-BR-3) and a non-neoplastic breast epithelial cell line (MCF10A) were purchased from Stem Cell Bank, Chinese Academy of Sciences. These cells were cultivated in RPMI1640 (Gibco, CA, USA) or DMEM (Gibco, CA, USA) supplemented with 10% fetal bovine serum (FBS; Gibco, CA, US) and incubated in a humidified atmosphere with 5% CO_2_ at 37 °C. All cell lines were propagated following the standard protocols from ATCC.

### RNA extraction and quantitative real-time RT-PCR

Total RNA was extracted using TRIZOL reagent (Invitrogen, Carlsbad, CA, USA) according to the manufacturer’s instructions, and then reverse transcribed into cDNA using a cDNA synthesis kit (Toyobo, Tokyo, JP). The quantitative real-time polymerase chain reaction (qRT-PCR) was performed using the THUNDERBIRD SYBR qPCR Mix (Toyobo, Tokyo, JP) according to the manufacturer’s instructions. ALL qRT-PCR were performed on the Applied Biosystems 7500 Real-Time PCR System ( (Bio-Rad, Hercules, CA, USA). GAPDH was measured as an internal control. The following gene-specific primers were used: GAPDH (F: 5′-GGTCGGAGTCAACGGATTTG-3′; R: 5′-ATGAGCCCCAGCCTTCTCCAT-3′). SNORA7B (F: 5′-TCCTGGGATCGCATCTGGA-3′; R: 5′-GGAATGGAATGGGTGCCTCT-3′). RPL32P3 (F: 5′-CGGCACCAGTCAGACCGATA-3′; R: 5′-CCTGCACCCGTGGTATAAAG-3′). Each sample was run in triplicate.

### Transfection of antisense oligonucleotides, small interfering RNAs, and plasmid DNAs

For functional studies, cells were transfected with the specific antisense oligonucleotides (ASO) flanked at both ends by locked nucleic acids or amido-bridged nucleic acids (AmNAs) targeting SNORA7B, siRNAs targeting RPL32P3, and SNORA7B-expression plasmids, which were synthesized and purchased from RiboBio. Cell transfection was performed with the Lipofectamine RNA iMAX (Life Technologies, Carlsbad, CA, USA) or Lipofectamine 3000 Reagent (Life Technologies, Carlsbad, CA, USA) according to the manufacturer’s protocol. The efficiency of transfection was confirmed by qRT-PCR. The sequence of each antisense oligonucleotides and siRNA is given in Additional file 1.

### Cell proliferation assay and colony formation assay

We utilized the colony formation and Cell Counting Kit-8 (CCK-8, Sigma, St Louis, MO, USA) assays to determine proliferative ability. For the colony formation assay, transfected MDA-MB-231 and BT-549 cells (2 × 10^3^ cells/well) were seeded in 6-well plates. After 10 days, cells were fixated with 4% paraformaldehyde (PFA) for 30 min and stained with 0.1% crystal violet for 30 min. Colonies were counted only if they included at least 50 cells. For the proliferation assay, the transfected cells (2 × 10^3^) were plated in 96-well plates and measured every 24 h using the CCK-8 reagent following the manufacturer’s instruction. The absorption was measured at 450 nm after adding the reagent and incubating for 2 h in a 37 °C incubator. All experiments were performed in triplicate.

### Cell migration and invasion ability analyses

Cellular migration and invasion assays were performed in a transwell cell culture chamber system and Matrigel invasion chamber system, respectively; both had a pore size of 8 mm according to the manufacturer’s instruction (Corning Costar, Cambridge, MA, USA). For migration assays, the transfected cells (8 × 10^5^ cells for MDA-MB-231 and 6 × 10^5^ cells for BT-549) were seeded in the upper chamber, which was placed into a 24-well plate filled with medium containing 10% FBS. Cells were incubated for 26 h (MDA-MB-231 cells) or 28 h (BT-549 cells) at 37 °C and 5% CO_2_ in an incubator. Then, after washing off cells that did not traverse the filter, cells adhering to the lower surface of the membrane were fixed with 4% PFA for 30 min, stained with 0.01% crystal violet for 30 min and photographed by a light microscope. For invasion assays, inserts coated with Matrigel matrix were used. The transfected cells (12 × 10^5^ cells for MDA-MB-231 and 10 × 10^5^ cells for BT-549) were added to the upper chamber, the medium in the lower chamber was supplemented with 20% FBS, and the chamber system was incubated for 24 h. The subsequent steps were similar to the migration assay. All experiments were performed at least three times.

### Cell apoptosis assay

Two days after infection, cells were harvested and double stained with annexin V conjugated to phycoerythrin and 7-aminoactinomycin (7-AAD) (Apoptosis Detection Kit-1, BD Pharmingen, San Diego, CA, USA). Apoptotic events were analyzed using FlowJo software. These experiments were repeated in triplicate.

### Statistical analysis

Data are expressed as the mean ± SD. The different gene expression levels of SNORA7B in tumor and healthy samples were analyzed using Wilcoxon signed-rank test, Mann–Whitney U test, and paired sample *t*-test. Spearman’s correlation analysis was used to determine correlation between SNORA7B and RPL32P3 expression level. Chi square test was used to access the relationship between SNORA7B expression and clinical characteristics. Survival curves were plotted by the Kaplan–Meier method and the log-rank tests. Both univariate and multivariate Cox proportional hazard models were applied to assess the relationship between effect of SNORA7B expression and survival. We used the Student’s *t*-test or one-way ANOVA to test the differences in expression between cell lines, the expression changes after transfection and all cell function assays. All *p*-values were two sided, and a *p*-value of 0.05 and less was considered statistically significant. Statistical analysis was performed with SPSS software version 19.0 (SPSS, Chicago, IL, USA). GraphPad Prism 6 (GraphPad Software, La Jolla, CA, USA) was used for plotting graphs.

## Result

### SNORA7B expression is frequently up-regulated in BC

**Fig. 1:**
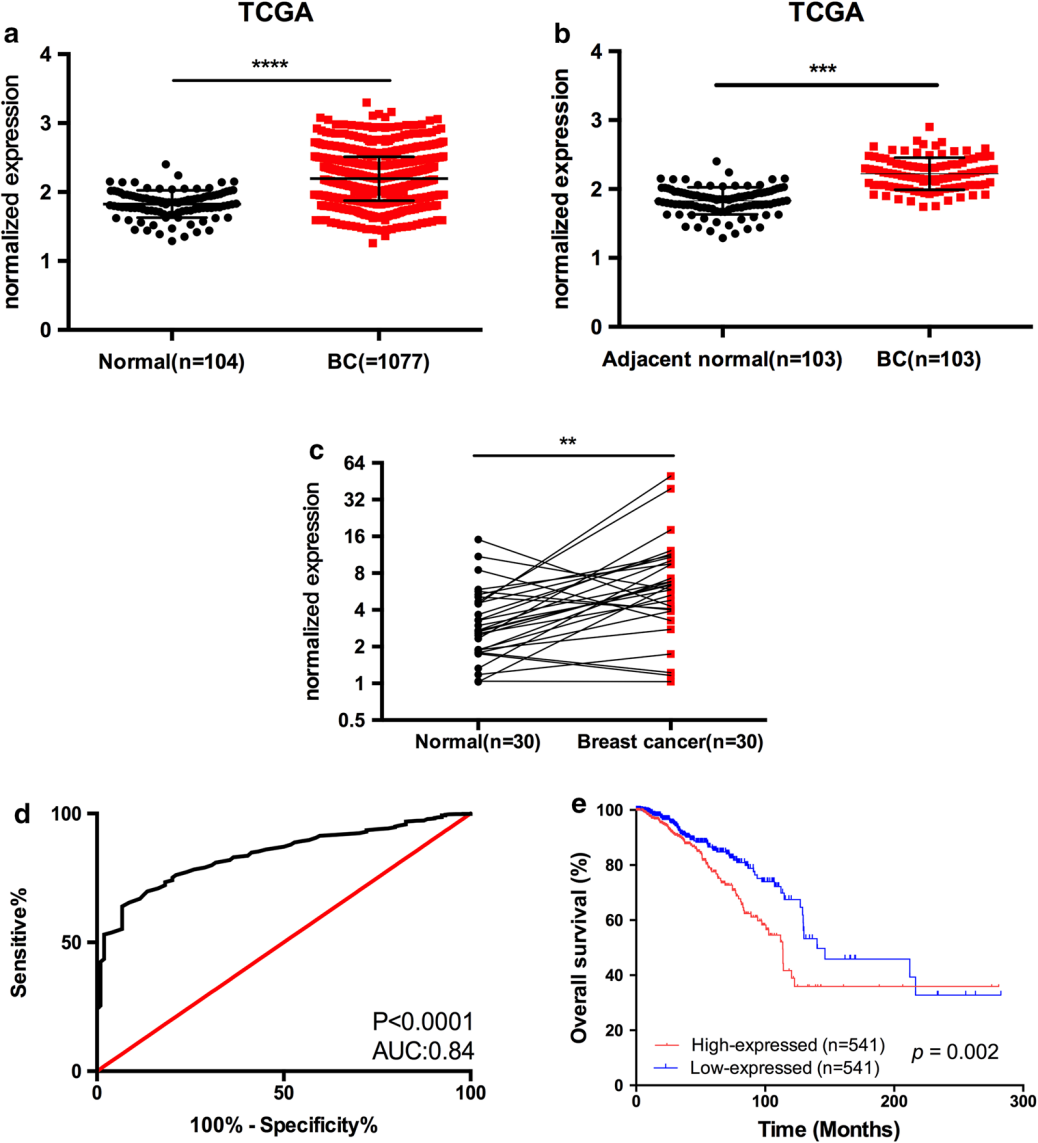
SNORA7B expression in breast cancer (BC) tissues and its clinical significance. **a**, **b** SNORA7B is upregulated in BC compared to both normal and adjacent normal tissues in TCGA dataset (p < 0.0001 and < 0.001, Mann–Whitney U test, Wilcoxon signed-rank test, respectively. **c** SNORA7B was overexpressed in breast cancer tissues compared to matched non-tumor tissues in our local cohort (p < 0.01, paired sample t-test). **d** Receiver operating characteristic (ROC) curve analysis of SNORA7B (p < 0.0001; AUC:0.84). **e** Kaplan–Meier plots of overall survival (OS). Patients with higher levels of SNORA7B exhibited poorer survival (p = 0.002, log-rank test)

We first analyzed snoRNA expression levels in BC from the publicly available TCGA database. To identify most pertinent and differentially expressed snoRNA candidates in BC, we selected differentially expressed snoRNAs that met the following criteria: adjusted *p* value < 0.05 and an absolute log fold change > 1.00. Accordingly, we identified SNORA7B, which was highly expressed in BC vs. normal samples in the TCGA dataset (Fig. 1a, b). To further investigate whether SNORA7B is up-regulated in BC, we assessed the mRNA expression levels of SNORA7B in 30 paired BC tissues and adjacent noncancerous breast tissues derived from fresh frozen samples via qRT-PCR analysis. As expected, SNORA7B expression was significantly higher in BC tissues than in normal tissues (Fig. 1c) (*p *= 0.002, Wilcoxon signed-rank test), which was consistent with the results in the TCGA dataset. Moreover, the receiver operating characteristic (ROC) curve analysis demonstrated that the expression of SNORA7B successfully discriminated BC from normal tissues (Fig. 1d). Taken together, these data suggest that SNORA7B is up-regulated in BC and has diagnostic potential in BC.

### SNORA7B expression and its relationship with the clinicopathologic features in BC

Next, we divided BC patients into 2 groups—those with high SNORA7B expression (n = 581) and those with low expression (n = 581)—and investigated the association between SNORA7B expression and different clinicopathological parameters in BC patients (Table 1). The results revealed that the expression of SNORA7B was positively correlated with older age (p = 0.003), bigger tumor size (p = 0.004), and lymph node metastasis (p = 0.045), whereas there were no statistically significant correlations with patient race, hormone receptor (HR) status, menopause status, HER2 status, distant metastasis, or AJCC stage.

**Table 1:** the relationship between SNORA7B expression and clinicopathological characteristics in TCGA cohort

Characteristics	Expression of SNORA7B		P
	Low (n = 541)	High (n = 541)	
Distant metastasis			0.695
M0	431	466	
M1	11	10	
Tumor stage (cm)			0.004*
≤ 2	160	119	
> 2	379	420	
Age			
Mean	57.19 ± 12.76	59.60 ± 13.57	0.003*
> 60	221	262	0.012
≤ 60	319	278	
HR status			0.787
Positive	414	401	
Negative	109	110	
HER2			0.392
Positive	86	95	
Negative	388	372	
Menopause status			0.13
Indeterminate	20	13	
Peri	24	16	
Post	329	365	
Pre	118	105	
Lymph node metastasis			0.045*
Yes	262	291	
No	272	236	
AJCC stage			0.058
I	107	73	
II	299	314	
III	116	129	
IV	11	10	
Race category			0.566
Asian	30	30	
Black or African American	102	80	
White	390	360	

* Statistically significant (p < 0.05)

To further evaluate the association between SNORA7B and prognosis of patients with BC, we performed Kaplan–Meier survival analysis and log-rank tests and revealed that high levels of SNORA7B expression resulted in significantly worse overall survival (OS) (Fig. 1e) (p = 0.002). This finding was further supported using both univariate and multivariate Cox proportional hazards model analyses (Table 2). The results of the univariate analysis showed that the SNORA7B expression [p = 0.003, HR (95% CI) 1.642 (1.188–2.271)], distant metastasis, tumor size, age, HR status, menopause status, LNM stage, and AJCC stage were significantly associated with OS. Likewise, the multivariate analysis revealed that when SNORA7B expression level, age, menopause status, AJCC stage, HR status were added to the analysis, high SNORA7B expression became an independent risk factor for OS [p = 0.029, HR (95% CI) 1.567 (1.047–2.346)] (Table 2). Together, our analysis indicated that SNORA7B overexpression in BC patients contributes to poor clinicopathologic features, further suggesting that SNORA7B might potentially be independent predictor of poor prognosis for BC.

**Table 2:** Univariate and multivariate Cox regression analysis of SNORA7B expression with regard to OS

Characteristics	Univariate analysis		Multivariate analysis	
	HR (95% CI)	P	HR (95% CI)	p
SNORA7B	1.642235 (1.188–2.271)	0.003*	1.567 (1.047–2.346)	0.029*
Distant metastasis	6.409 (3.792–10.831)	< 0.001*		
Tstage	1.46 (1.199–1.778)	< 0.001*		
Age	1.029 (1.017–1.042)	< 0.001*	1.047 (1.025–1.070)	< 0.001*
HR status	0.653 (0.454–0.94)	0.022*	0.488 (0.315–0.756)	0.001*
Her2	1.147 (0.696–1.891)	0.590		
Menopause status	1.434 (1.112–1.849)	0.005*	0.887 (0.614–1.280)	0.521
LNM	1.641 (1.383–1.946)	< 0.001*		
AJCC stage	2.203 (1.769–2.743)	< 0.001*	2.612	< 0.001*
Race category	0.952 (0.683–1.326)	0.769		

* Statistically significant (p < 0.05)

### SNORA7B, rather than its host gene RPL32P3, is overexpressed in BC cell lines

**Fig. 2:**
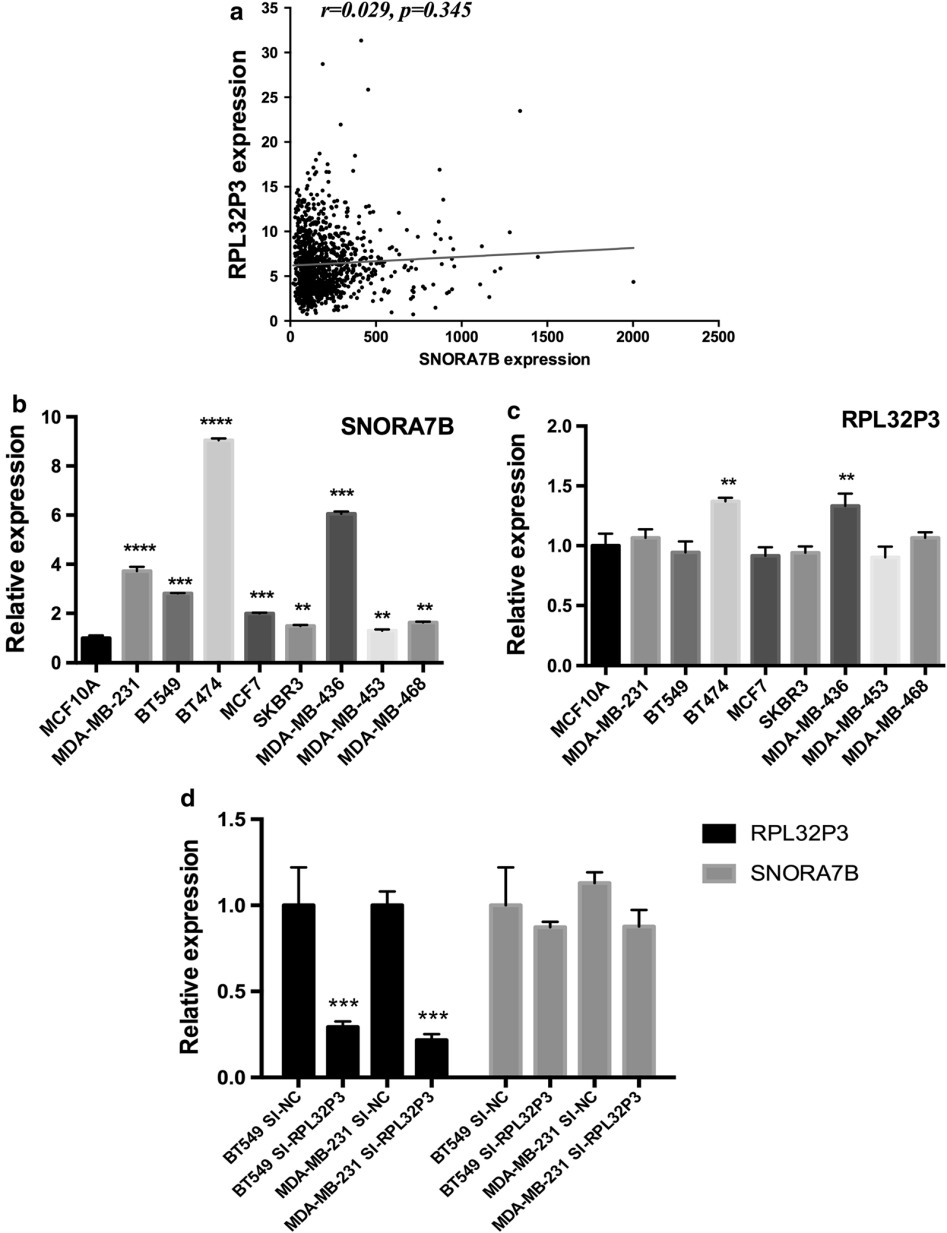
SNORA7B rather than its host gene RPL32P is upregulated in BC cell lines. **a** The correlation between the expression of SNORA7B and RPL32P was not observed (p = 0.345 > 0.05, Pearson correlation analysis). **b**, **c** The expression levels of SNORA7B and RPL32P3 RNA in 8 BC cell lines and a control cell line (MCF10A) via qRT-PCR. **d** Expression of RPL32P3 declined while that of SNORA7B did not change significantly in MDA-MB-231 and BT-549 cell lines after transfection with siRNA targeting RPL32P3. (**p < 0.01; ***p < 0.001; ****p < 0.0001 (Student’s t test).)

Based on the clinical significance of SNORA7B in BC, we next investigated the oncogenic roles of SNORA7B in BC cell lines. SNORA7B is located as an intron in the RPL32P3 gene. To explore the potential relationship between SNORA7B and RPL32P3 and to identify the most pertinent cell for experiments, we firstly analyzed the expression levels of SNORA7B and RPL32P3 in 1082 BC patients and found that there was no correlation (r = 0.029, p = 0.345) between SNORA7B and its host gene (Fig. 2a). Next, the comparative mRNA expression levels of both genes were examined in BC cell lines by qRT-PCR. The results demonstrated that SNORA7B was up-regulated in all BC cell lines compared with its expression in normal breast cell line MCF10A, while RPL32P3 was overexpressed in only two cancer cell lines compared to MCF10A (Fig. 2b, c). Furthermore, SNORA7B was statistically unchanged after MDA-MB-231 and BT-549 cells were transfected with siRNA against RPL32P3 (Fig. 2d). We also tested expression levels of RPL32P3 after knocking down SNORA7B, and found that the RPL32P3 expression level was not affected as expected (Additional file 2). These results imply that SNORA7B rather than its host gene is frequently overexpressed in breast cancer cells.

### SNORA7B knockdown decreases proliferation and colony formation

**Fig. 3:**
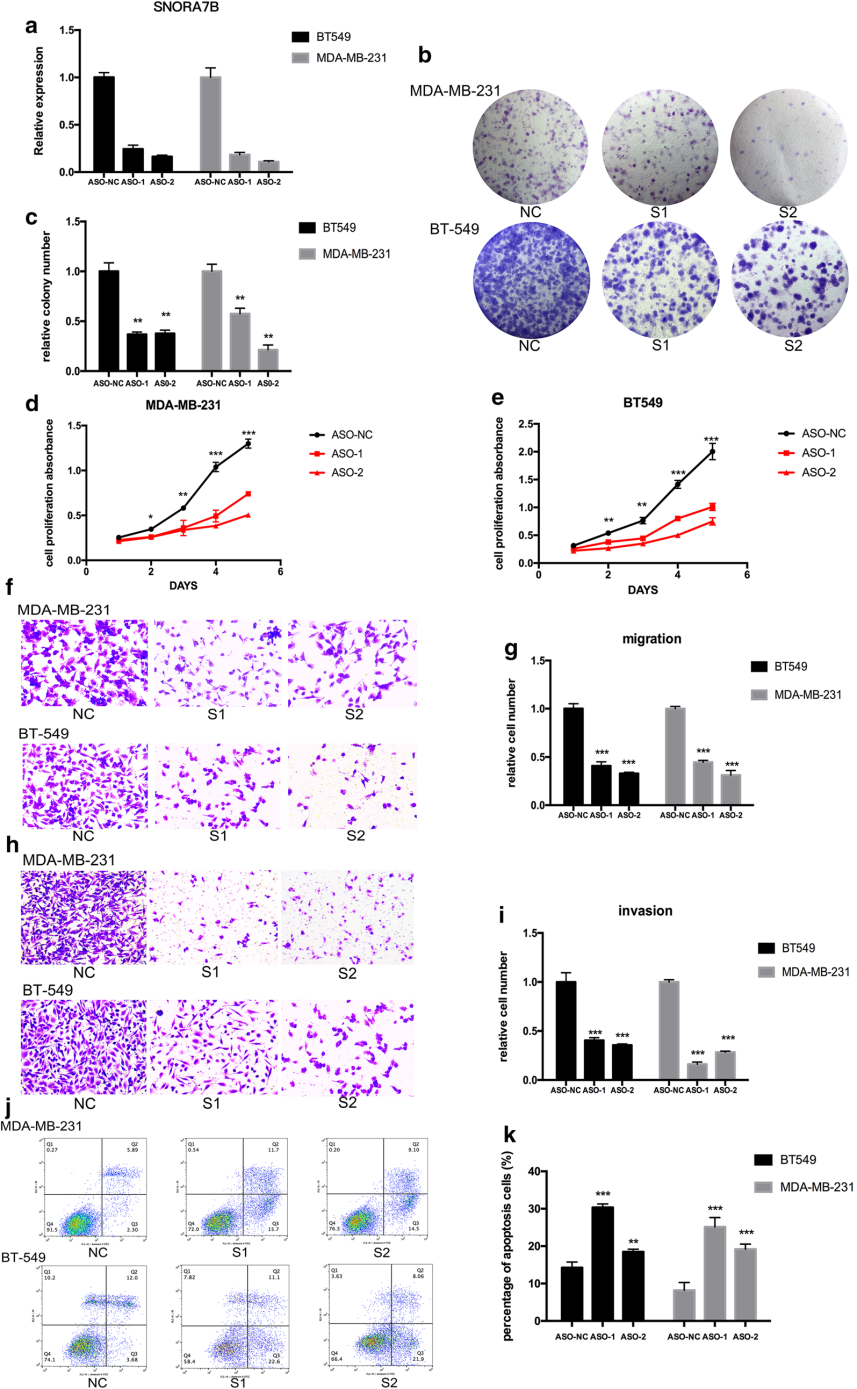
SNORA7B knockdown inhibits tumorigenicity and promotes apoptosis in BC cells. **a** qRT-PCR analysis of SNORA7B expression of MDA-MB-231 and BT-549 cell lines treated with specific ASOs against SNORA7B. **b**, **c** Colony formation assays were performed to assess the cell growth after SNORA7B knockdown. **d**, **e** CCK8 assays were used to assess the proliferative capacity of BC cells transfected with anti-SNORA7B-ASOs. **f**, **g** Effect of SNORA7B downregulation on migration was examined by migration assays. **h**, **i** Invasion ability of BC cells was accessed by invasion assays. **j** Annexin V/PI (propidium iodide) staining was performed in MDA-MB-231 and BT-549 cell lines with suppression of SNORA7B. **k** Percentage of apoptosis (early apoptotic cells and late apoptotic cells) in SNORA7B suppressed BC cells. (These experiments were repeated independently for three times. *p < 0.05; **p < 0.01; ***p < 0.001; ****p < 0.0001 (Student’s t test).)

Sequentially, we performed loss-of-function analysis to explore the biological effects of SNORA7B dysregulation. MDA-MB-231 and BT-549 cell lines were selected for inhibition of SNORA7B via transfection with specific ASOs targeting SNORA7B. The qRT-PCR results showed that the expression of SNORA7B was successfully decreased (> 70%) in both cell lines (p < 0.001; Fig. 3a). Next, we performed the colony formation and CCK-8 assays to investigate the role of SNORA7B on cell growth and proliferation. As shown in Fig. 3b, c, the MDA-MB-231 and BT-549 transfected with anti-SNORA7B-ASOs formed less colonies compared with the control group (p < 0.01). Consistent with the colony formation assay, the CCK-8 assay showed that the proliferative capacity of the cell lines transfected with anti-SNORA7B-ASOs was significantly attenuated (p < 0.001; Fig. 3d, e). These data indicated that down-regulation of SNORA7B could suppress the proliferation and growth abilities of MDA-MB-231 and BT-549 cancer cells.

### SNORA7B knockdown impairs the migration and invasion

To further investigate whether the knockdown of SNORA7B expression could regulate BC migration and invasion abilities, we carried out migration and invasion assay as described above in MDA-MB-231 and BT-549 cell lines. Cancer cells with SNORA7B knockdown displayed much fewer migrating cells though the membrane after 26 h than cells transfected with negative control (p < 0.001; Fig. 3f, g). Consistently, the invasion assays showed that SNORA7B knockdown resulted in a dramatic decrease in the invasion capacity of the cells compared with the control group (p < 0.001; Fig. 3h, i). Thus, SNORA7B knockdown not only promotes cell proliferation but also significantly inhibits tumor metastasis in breast cancer cell lines. Together, these results were consistent with our clinical findings.

### SNORA7B knockdown induces apoptosis

To explore whether the cell proliferation and metastasis function of SNORA7B is associated with apoptosis, we carried out 7-AAD and annexin V staining in MDA-MB-231 and BT-549 cells after different treatments to evaluate cell death and apoptosis by flow cytometry. We found that the percentage of apoptotic cells were markedly increased in anti-SNORA7B-ASO-transfected MDA-MB-231 and BT-549 cells compared with control groups (p < 0.01; Fig. 3j, k). These findings suggested that SNORA7B knockdown inhibits proliferation and metastasis by triggering cell apoptosis.

### Overexpression of SNORA7B greatly promotes BC cell tumorigenesis

**Fig. 4:**
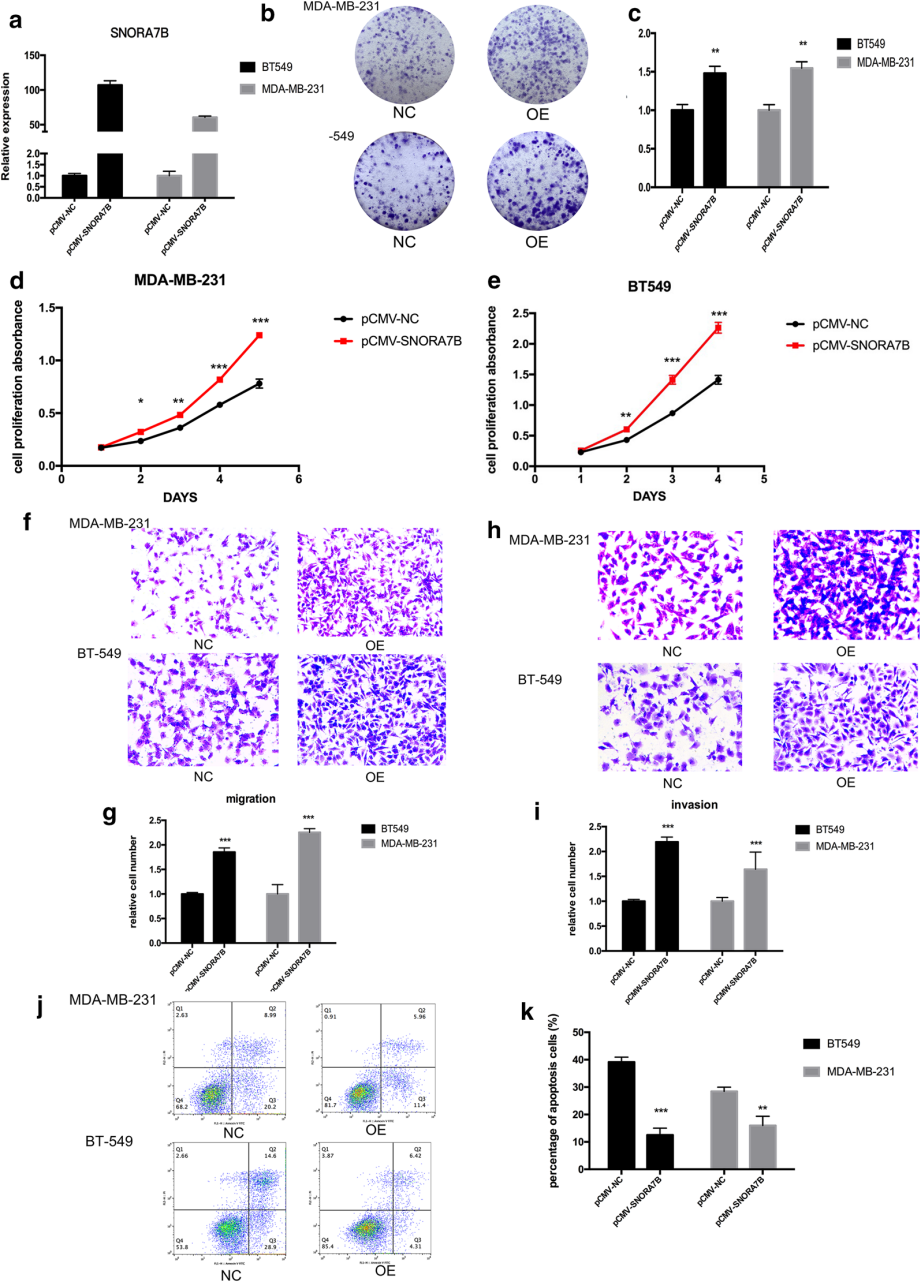
SNORA7B overexpression promotes tumorigenicity and inhibits apoptosis in BC cells. **a** Efficiency of SNORA7B overexpression in MDA-MB-231 and BT-549 cell lines by qRT-PCR after transfection with pCMV-SNORA7B and pCMV-NC. **b**, **c** SNORA7B overexpression in MDA-MB-231 and BT-549 cell lines results in increased colonies. **d**, **e** SNORA7B overexpression increased proliferative capacity of MDA-MB-231 and BT-549 cell lines. **f**, **g** SNORA7B overexpression in both cell lines resulted in increased migration capacity. **h**, **i** Invasion capacity enhanced in both cell lines transfected with SNORA7B overexpression plasmid. **j**, **k** SNORA7B overexpression reduced the percentage of apoptotic cells in both BC cell lines. (These experiments were repeated independently three times. *p < 0.05;**p < 0.01;***p < 0.001;****p < 0.0001 (Student’s *t* test)

To support our findings from loss-of-function experiments, we further explored the role of SNORA7B overexpression in MDA-MB-231 and BT-549 cells. Both cell lines were transfected with pCMV-SNORA7B and pCMV control. After 48 h of transfection, the expression level of SNORA7B was substantially enhanced by approximately 100- and 60-fold in BT-549 and MDA-MB-231 cells, respectively, as shown by qRT-PCR (p < 0.001; Fig. 4a). The proliferation (colony formation) of pCMV-SNORA7B transfected cells was significantly increased compared with the pCMV controls (p < 0.01; Fig. 4b, c). Consistently, pCMV-SNORA7B-cells displayed higher cell growth rate than pCMV controls (p < 0.001, Fig. 4d, e). Additionally, in cell migration and invasion assays, we found a significant increase in the number of cancer cells that penetrated the porous filter in pCMV-SNORA7B-cells compared with cells transfected with pCMV control (p < 0.001; Fig. 4f–i) and the population of pCMV-SNORA7B cells showed a significant decrease in early and late apoptotic cells (p < 0.01; Fig. 4j, k). Together, our observations from the gain-of-function experiments in both cell lines were consistent with the loss-of-function analysis, providing robust evidence that SNORA7B could function as an oncogene in BC.

## Discussion

SnoRNAs are a type of noncoding RNA reported to play important regulatory roles in several physiological and pathological events [21–23]. A growing volume of evidence indicates that multiple snoRNAs are involved in various cancers [24–28]. In light of the potential value of snoRNAs as biomarkers and therapeutic targets, as well as to further explore the potential molecular mechanisms underlying cancer formation and development, we identified and investigated potential snoRNAs involved in BC. In this study, we firstly screened for specific snoRNAs using the publicly available TCGA database. Our analysis of the database indicated that the expression of SNORA7B was prominently up-regulated in BC compared with normal tissues, which was also verified in our breast cancer clinical specimens and cell lines. Intriguingly, we also confirmed that SNORA7B expression was positively associated with some key clinicopathological parameters, such as age, tumor size, and lymph node metastasis. Moreover, patients with high expression of SNORA7B showed much worse prognosis of BC. These results suggested that the SNORA7B might play an oncogenic role in breast cancer progression. Thus, SNORA7B, which is positioned on the intron of RPL32P3 gene and belongs to the H/ACA snoRNAs for sequence-specific pseudouridylation of other RNAs, was selected for further functional examination.

In most cases, the transcription of the host gene determines the levels of intron encoded snoRNA genes [29]. Thus, considering the possibility that RPL32P3 also might play a role in BC, we examined the expression of RPL32P3 in TCGA database and BC cell lines. Interestingly, RPL32P3 exhibited only little difference in RNA expression level between BC cell lines and normal breast cell line, and no correlation was found between transcriptional levels of SNORA7B and RPL32P3. Moreover, we also evaluated the expression of SNORA7B following the knockdown of RPL32P3 and found that its expression was only slightly and insignificantly altered. These results suggested that the function of SNORA7B might be independent of its host gene.

We then performed a series of gain- and loss-of- function analyses in vitro to further determine the functional significance of SNORA7B in BC cell lines. By using specific anti-SNORA7B-ASOs to suppress SNORA7B expression, we elucidated that SNORA7B knockdown exhibited a strong capacity to promote cell apoptosis, which resulted in the inhibition of cell growth, proliferation, migration, and tumor invasion abilities of the BC cells. The oncogenic effect of SNORA7B in BC was subsequently confirmed with SNORA7B overexpression, which showed consistent results with that from the loss-of-function experiments.

Previous study and computational analysis indicated that there are several isoforms of SNORA7 [23]. Among these paralogues, SNORA7B and SNORA7A are over 98% identical and target the same 28S rRNA pseudouridylation sites [30, 31]. Zhang et al. [32] demonstrated that both SNORA7B and SNORA7A could promote the self-renewal of human umbilical cord blood-derived mesenchymal stem cells (uMSCs). The SNORA7B gene is located on chromosome 3q21 as an intron in the RPL32P3 gene. Interestingly, genomic alterations in the 3q21 locus have been observed in leukemia [33, 34], colorectal cancer [35], prostate cancer [36], and breast cancer [37–39]. Each class of snoRNAs interacts with a specific set of highly conserved proteins to form the well-defined C/D box and H/ACA box small nucleolar ribonucleoproteins (snoRNPs). The H/ACA box snoRNAs are associated with four proteins including DKC1, GAR1, NHP2, and NOP10 to catalyze a certain pseudouridylation site of 18S or 28S rRNA [8, 40]. Considering that cancer cells often show perturbation at the translation level, snoRNAs and snoRNPs are likely to contribute to tumorigenesis through effects on ribosomes and protein translation. Zhang et al. [32] also showed that SNORA7A functioned by inducing snoRNP formation by binding DKC1 and subsequently catalyzing pseudouridines in 28S rRNA. Since SNORA7B and SNORA7A target the same 28S rRNA pseudouridylation sites, it is likely that SNORA7B may also act through snoRNP to regulate the tumor behavior in BC.

The present study still had several limitations. First, this study only conducted in vitro experiments. Therefore, the effects of SNORA7B in BC need to be verified in vivo experiments. Second, the mechanism of SNORA7B oncogenic role needs to be further explored. Despite these limitations, this study is the first to identify the oncogenic role of SNORA7B and show that it promotes BC cell growth, proliferation, invasion, and migration through decreased apoptosis of BC cells in vitro. Furthermore, the expression of SNORA7B, which was significantly up-regulated in BC, may have diagnostic potential and present a useful prognostic molecular marker of BC.

## Conclusions

In summary, we found that expression level of SNORA7B increased in BC tissues compared to that in non-tumor tissues. Further, SNORA7B expression level was positively correlated with poor survival time and worse clinicopathologic parameters. SNORA7B impaired apoptosis to promote BC cell growth, proliferation, migration, and invasion. Here, for the first time, we identified that SNORA7B functions as an oncogene in BC and may have diagnostic potential and sever as a potential prognostic biomarker for BC.

## Additional files

**Additional file 1.** knockdown oligos used in the article.

**Additional file 2.** Supplementary figure and legends: the RNA expression level of RPL32P3.

## Abbreviations

BC: breast cancer
snoRNAs: small nucleolar RNAs
SNORA7B: H/ACA box small nucleolar RNA 7B
RPL32P3: ribosomal protein L32 pseudogene 3
TCGA: The Cancer Genome Atlas
ROC: the receiver operating characteristic
HR status: hormone receptor status
HER-2: human epidermal growth factor receptor-2
OS: overall survival
qRT-PCR: quantitative real-time polymerase chain reaction
HR: hazard ratio
CI: confidence interval
ASO: antisense oligonucleotides
PFA: paraformaldehyde
