# Supplementary material for: H/ACA box small nucleolar RNA 7B acts as an oncogene and a potential prognostic biomarker in breast cancer
Source: Cancer Cell Int. 2019 May 9;19:125. doi: 10.1186/s12935-019-0830-1 (PMC6509762; doi:10.1186/s12935-019-0830-1)
Supplement: Supplementary file 2 — Additional file 2. Supplementary figure and legends: the RNA expression level of RPL32P3. [file 12935_2019_830_MOESM2_ESM.docx]

**Supplementary figure and legends: the RNA expression level of RPL32P3**


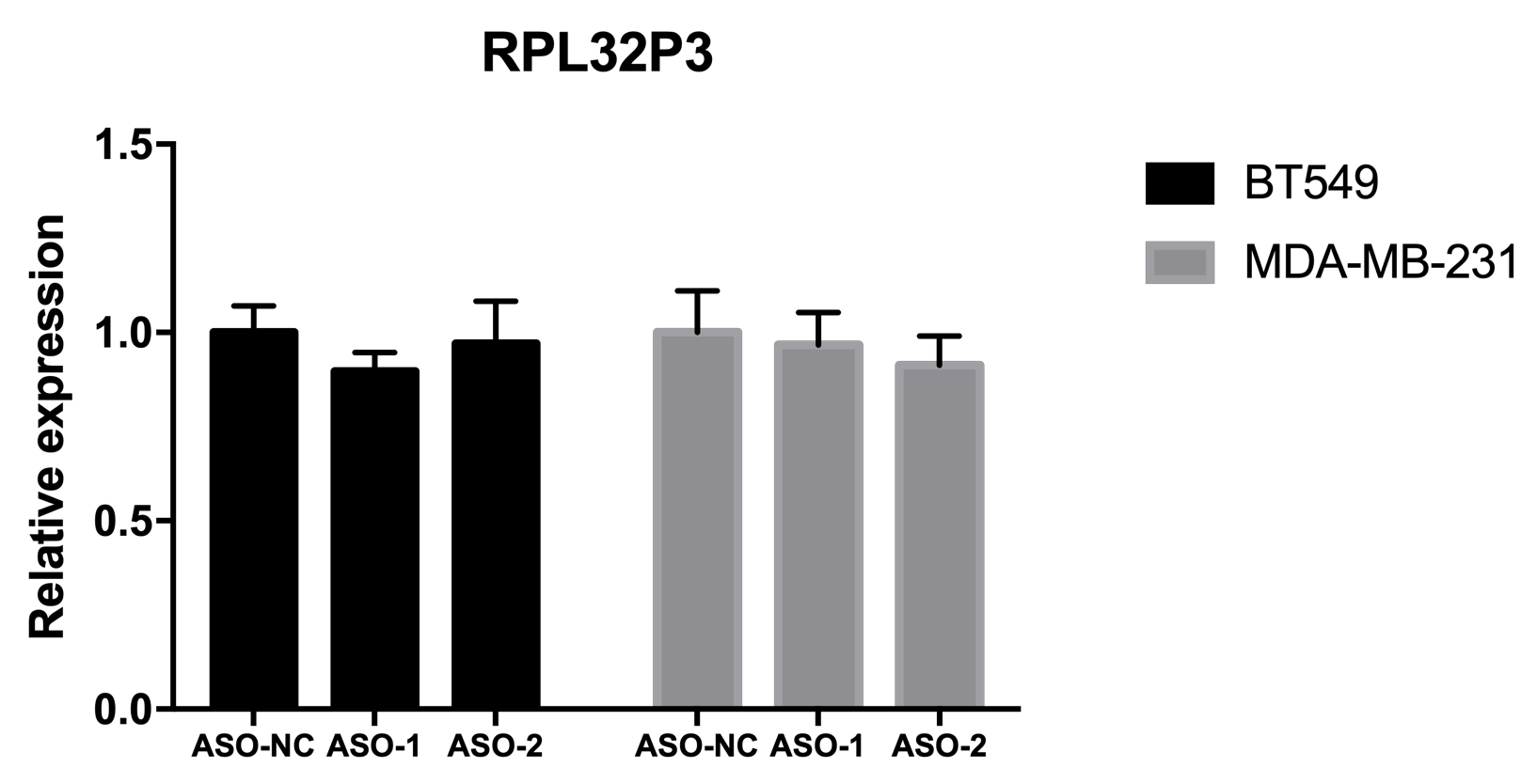


**Fig. supplement SNORA7B knock down did not affect the expression level of RPL32P3.** qRT-PCR analysis of RPL332P3 expression in MDA-MB-231 and BT-549 cell lines treated with specific ASOs against SNORA7B.
